# Circulating mid-regional proadrenomedullin is a predictor of mortality in patients with COVID-19: a systematic review and meta-analysis

**DOI:** 10.1186/s12879-023-08275-z

**Published:** 2023-05-08

**Authors:** Na Wang, Lushan Liu, Wei He, Na Shang, Junyu Li, Zhou Qin, Xiaoxia Du

**Affiliations:** 1 Emergency department of China Rehabilitation Research Center, Capital Medical University, no.10 Jiaomen north Street, Fengtai District, Beijing, 100068 China; 2 Department of neurorehabilitation of China Rehabilitation Research Center, Capital Medical University, no.10 Jiaomen north Street, Fengtai District, Beijing, 100068 China

**Keywords:** Mid-regional Proadrenomedullin, COVID-19, Mortality, Meta-analysis

## Abstract

**Background:**

Although there is increasing understanding of the changes in the laboratory parameters of Coronavirus disease 2019 (COVID-19), the correlation between circulating Mid-regional Proadrenomedullin (MR-proADM) and mortality of patients with COVID-19 is not fully understood. In this study, we conducted a systematic review and meta-analysis to evaluate the prognostic value of MR-proADM in patients with COVID-19.

**Methods:**

The PubMed, Embase, Web of Science, Cochrane Library, Wanfang, SinoMed and Chinese National Knowledge Infrastructure (CNKI) databases were searched from 1 January 2020 to 20 March 2022 for relevant literature. The Quality Assessment of Diagnostic Accuracy Studies (QUADAS-2) was used to assess quality bias, STATA was employed to pool the effect size by a random effects model, and potential publication bias and sensitivity analyses were performed.

**Results:**

14 studies comprising 1822 patients with COVID-19 met the inclusion criteria, there were 1145 (62.8%) males and 677 (31.2%) females, and the mean age was 63.8 ± 16.1 years. The concentration of MR-proADM was compared between the survivors and non-survivors in 9 studies and the difference was significant (P < 0.01), I^2^ = 46%. The combined sensitivity was 0.86 [0.73–0.92], and the combined specificity was 0.78 [0.68–0.86]. We drew the summary receiver operating characteristic (SROC) curve and calculated the area under curve (AUC) = 0.90 [0.87–0.92]. An increase of 1 nmol/L of MR-proADM was independently associated with a more than threefold increase in mortality (odds ratio (OR) 3.03, 95% confidence interval (CI) 2.26–4.06, I^2^ = 0.0%, P = 0.633). The predictive value of MR-proADM for mortality was better than many other biomarkers.

**Conclusion:**

MR-proADM had a very good predictive value for the poor prognosis of COVID-19 patients. Increased levels of MR-proADM were independently associated with mortality in COVID-19 patients and may allow a better risk stratification.

**Supplementary Information:**

The online version contains supplementary material available at 10.1186/s12879-023-08275-z.

## Introduction

Coronavirus disease-19 (COVID-19) is a clinical syndrome caused by the novel Severe Acute Respiratory Syndrome Coronavirus 2 (SARS-CoV-2). The COVID-19 pandemic spread rapidly with more than 535 million cases and 6.3 million deaths. COVID-19 is a global problem and a significant cause of death. Therefore, accurate evaluation of patients’ prognosis is very important. Many inflammatory markers [1–5] and critical illness scores [6–10] are used to predict the prognosis and mortality of patients with COVID-19 infection.

The emergence of an increasing number of biomarkers may provide a new way to improve the accuracy of prognosis simply and quickly. Mid-regional Proadrenomedullin (MR-proADM) is a peptide produced by multiple tissues that is used to stabilize microcirculation and protect endothelial function [11–14]. Previous studies have shown that with the deterioration of microcirculation integrity and capillary leakage, the concentration of MR-proADM increases, reflecting the early development stage of organ dysfunction [14, 15]. Therefore, early evaluation of microcirculation function may provide a valuable tool to evaluate disease progression and treatment effect [15].

Some studies were devoted to investigate the relationship between MR- proADM and the outcome of patients with COVID-19 [16–33]. However, their sample sizes were small, and the conclusions were inconsistent. Using a meta-analytic method, the aim of the present study was to provide an overview of the concentration of the MR-proADM between the survivors and non-survivors and to explore the predictive value of the MR-proADM for the mortality of the hospitalized patients with COVID-19.

## Methods

This meta-analysis was performed in accordance with the Preferred Reporting Items for Systematic Reviews and Meta-Analyses (PRISMA) guidelines [34]. The review protocol was registered on PROSPERO (CRD42022308638).

## Search strategy

**Fig. 1:**
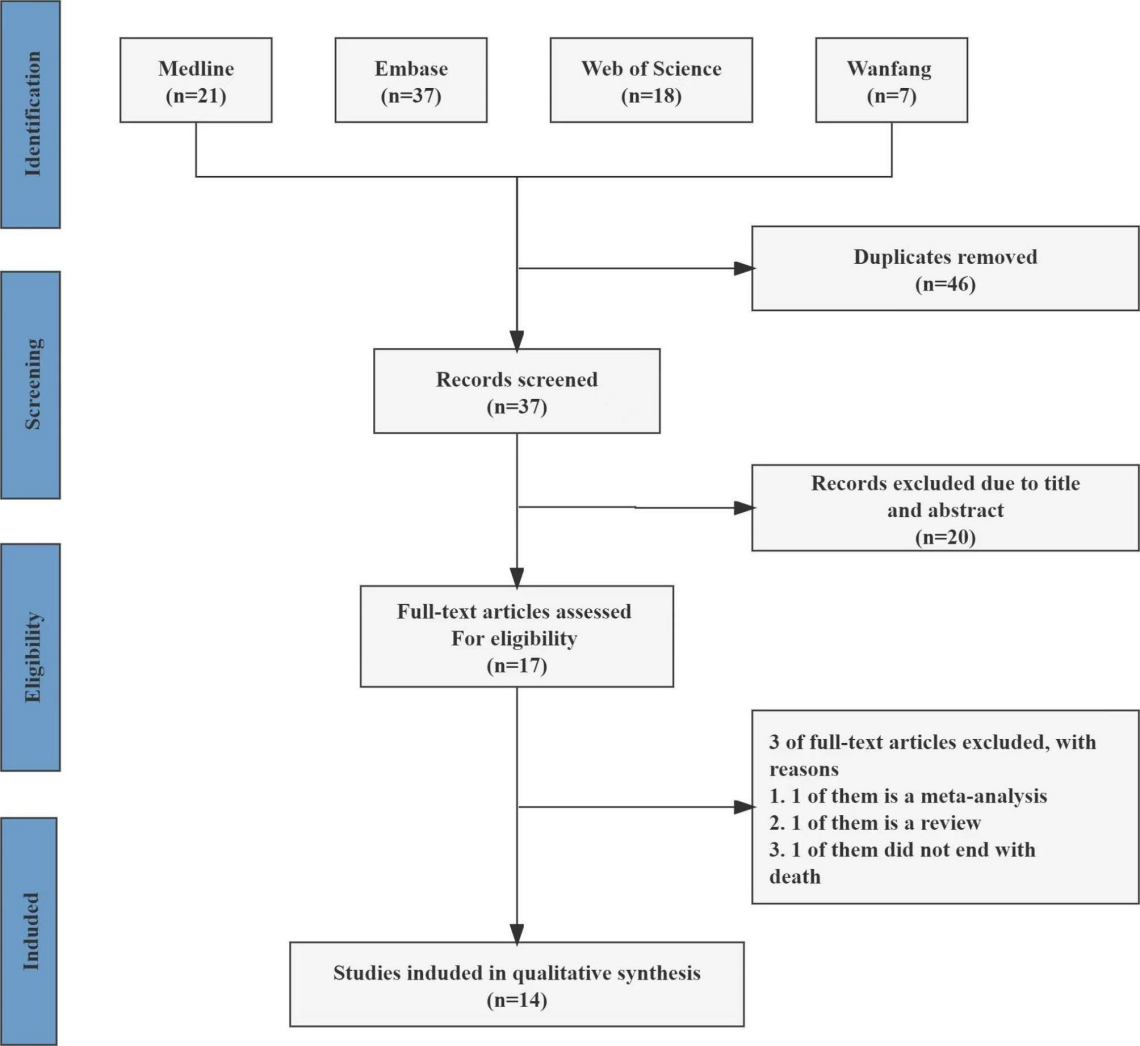
Research screening flowchart

The systematic literature search was conducted in PubMed, Embase, Web of Science, the Cochrane Library, Wanfang, SinoMed and Chinese National Knowledge Infrastructure (CNKI) for studies published from 1 January 2020 to 20 March 2022. The following search terms were used: “MR-proADM” OR “MR-pro-adrenomedullin” OR “midregional pro-adrenomedullin” OR “MR-pro-ADM” and “COVID-19” OR “SARS-CoV-2 Infection” OR “2019 Novel Coronavirus Disease” OR “2019 Novel Coronavirus Infection” OR “2019-nCoV Disease”. There was no restriction on the language of the records. See Fig. 1 for details.

## Selection and eligibility criteria

All prospective and retrospective studies of MR-proADM and outcomes in patients with COVID-19 were included, and there were no restrictions on age, sex, setting of treatment, severity of disease, sample size, etc. Death was the primary endpoint, and studies that did not include death outcomes were excluded.

## Data extraction

Two authors independently extracted data into a standardized data collection form. Discrepancies were resolved by a third party. The following data were extracted from each study: first author, publication year, country, study period, study design, setting, population, number, age, male ratio, clinical outcomes, levels of MR-proADM, mortality rate, AUC for death, cut-off value, sensitivity and specificity. See additional files for detail.

## Risk of bias assessment

**Fig. 2:**
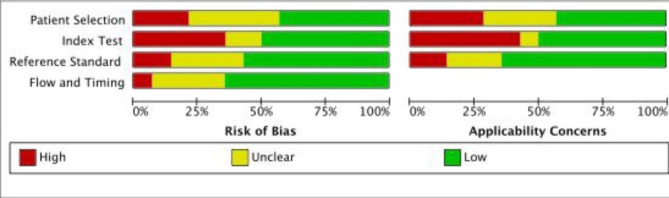
Assessment of risk of bias for the studies

The quality of the included studies was assessed by two independent assessors using the Quality Assessment of Diagnostic Accuracy Studies (QUADAS-2) which consists of four key domains: patient selection, index test, reference standard and flow and timing (Fig. 2). Each was assessed in terms of risk of bias and the first three in terms of concerns regarding applicability. Signaling questions were included to assist in judgements about the risk of bias.

## Data synthesis and statistical analysis

The main analysis was to determine the predictive ability of MR-proADM on COVID-19 mortality. The mean and standard deviation (SD) of the MR-proADM values were included in the pooled analysis. A random model was used to calculate and separately pool the concentration of MR-proADM in the survivors and non-survivors, and the weighted mean difference (WMD) and 95% confidence interval (95% CI) of survivors and non-survivors were estimated. When the mean and standard deviation could not be obtained, conversion calculations were carried out according to the sample size, median and interquartile range (IQR) as suggested by Hozo et al. [35].

The heterogeneity between the different studies was presented as I^2^ and P values for the I^2^ statistic were derived from the chi-square distribution of Cochran’s Q test. The standard for the category of heterogeneity was defined as I^2^ > 50%. We used STATA 16.0 to calculate the sensitivity and specificity of MR-proADM and performed the summary receiver operating characteristic (SROC) curve of MR-proADM for mortality. Public bias was examined by Egger’s test.

All statistical analyses were performed using RevMan version 5.4 (the Cochrane Collaboration) and STATA 16.0 software (StataCorp, College Station, Texas, USA). P values less than 0.05 were considered statistically significant.

## Results

### Literature search

We found 83 relevant studies from seven databases, Medline (21), Embase (37), Web of Science (18), Wanfang (7) and the other three databases contain zero records. Endnote software was used to delete duplications, and 37 articles remained. By checking the titles and abstracts, another 20 articles were excluded. Three of the remaining 17 articles did not meet the inclusion criteria, and finally 14 articles were ultimately included. The literature search and selection process are depicted in the PRISMA flow diagram (Fig. 1).

The included studies were published between 2021 and 2022. We looked for an association between MR-proADM and the mortality of patients with COVID-19. A total of 1822 patients met the inclusion criteria, including 1145 (62.8%) males and 677 (31.2%) females, aged 63.8 ± 16.1 years. Eight of the studies were from Italy, three were from Spain and others were from the Netherlands, Switzerland and United Kingdom. Three studies were conducted in hospital settings, three in the intensive care unit (ICU), three in the emergency department, one in a COVID center, one in an intermediate medical care unit (IMCU), one in a medium intensity-of-care COVID-19 department, one in an acute national health service (NHS) setting and the remaining study site was not determined. Nine studies used a prospective study design, and five studies were retrospective studies. Of these studies, 12 were single-center studies and two were multicenter studies. Three studies reported 28-day mortality, six reported in-hospital mortality, four reported 30-day mortality and one reported 90-day mortality. We summarized the characteristics of the included studies in Supplementary Tables 1, 2, 3.

### Meta-analysis of the effects of MR-proADM on mortality

**Fig. 3:**
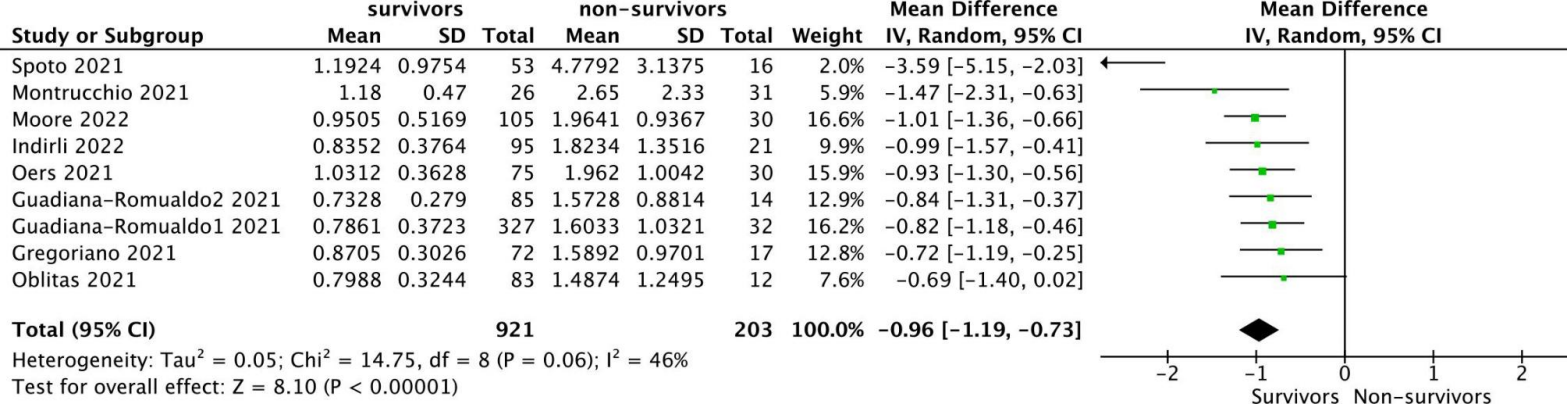
Forest plots showing WMD with 95% CI for the concentration of MR-proADM Comparing survivors and non-survivors in a random-effect model. CI, confidence interval; WMD, weighted mean difference

**Fig. 4:**
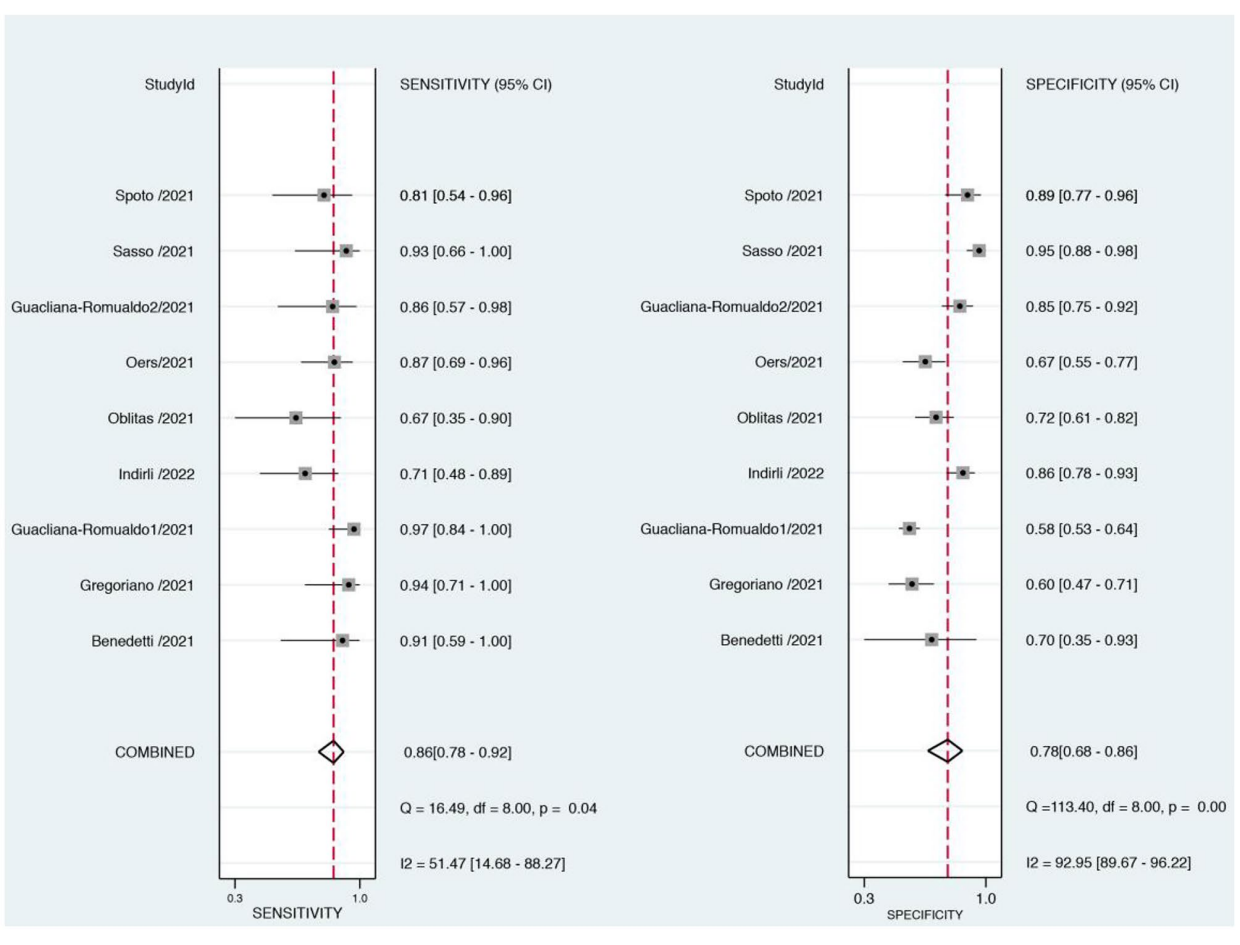
Forest plot of the sensitivity and specificity of MR- proADM to predict mortality in COVID-19 patients. CI, confidence interval

Of 14 studies, 10 studies described the specific concentration of MR-proADM in the survivors and non-survivors. Therefore, the concentration of MR-proADM was compared between the survivors and non-survivors in 10 studies and the difference was significant (P < 0.01), I^2^ = 79%. Due to the obvious heterogeneity, we looked for the source of heterogeneity and found that after removing the first study [24], the heterogeneity of the results decreased significantly. Finally, nine studies were included, and there was a significant difference in the concentration of MR- proADM between the survivors and the non-survivors (P < 0.01), I^2^ = 46% (Fig. 3). STATA was used to calculate the combined sensitivity and specificity, the combined sensitivity was 0.86 [0.73–0.92], I^2^ = 51.47%, and the combined specificity was 0.78 [0.68–0.86], I^2^ = 92.95% (Fig. 4).

**Fig. 5:**
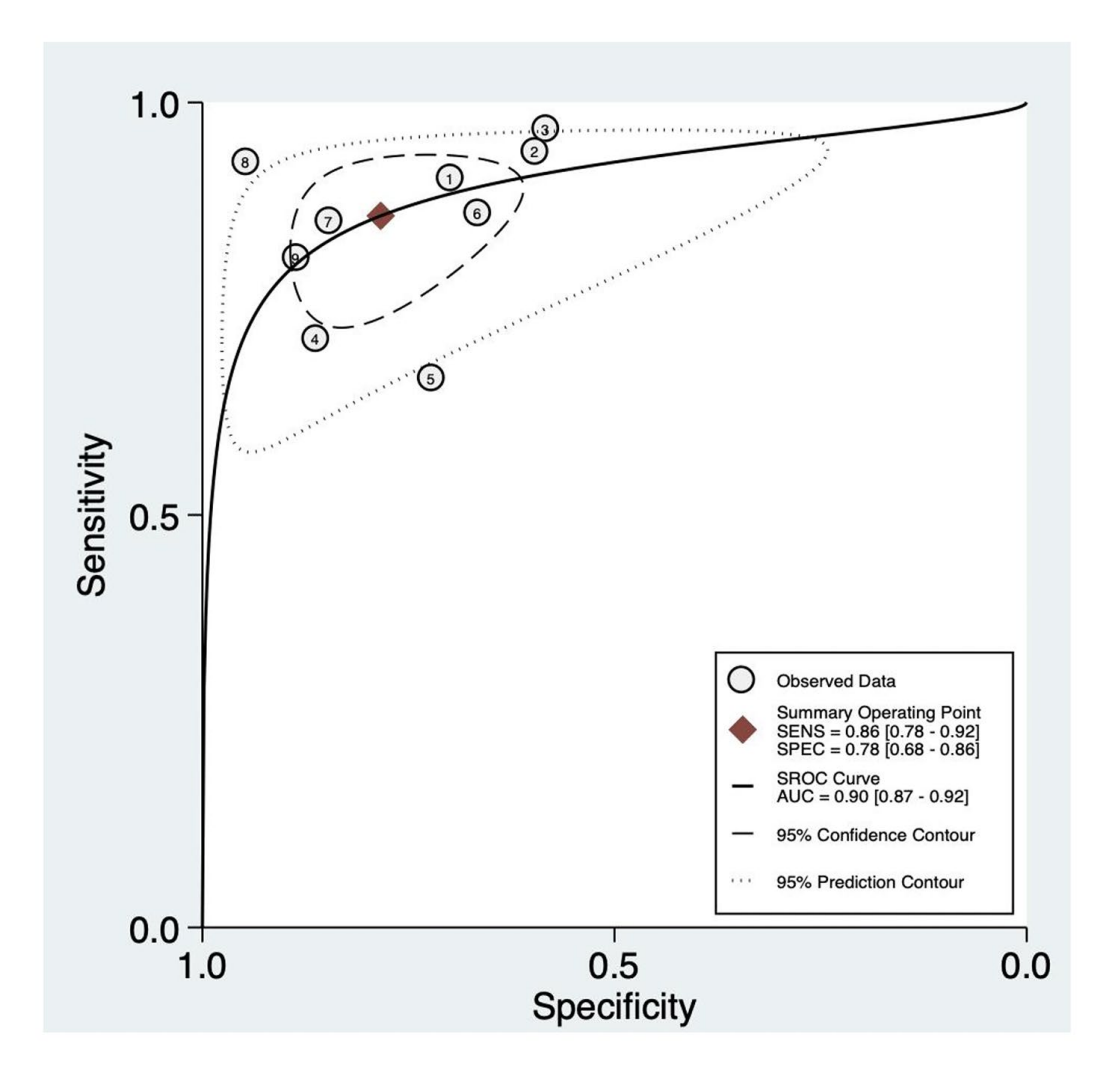
Summary receiver operating characteristics (SROC) curve for the included studies. SENS, sensitivity; SPEC, specificity; AUC, area under the curve

To evaluate the predictive ability of MR- proADM for the mortality of patients with COVID-19, we drew the SROC curve, and calculated the AUC = 0.90 [0.87–0.92] (Fig. 5).

### Odds ratios (OR) and hazard ratios (HR)

**Fig. 6:**
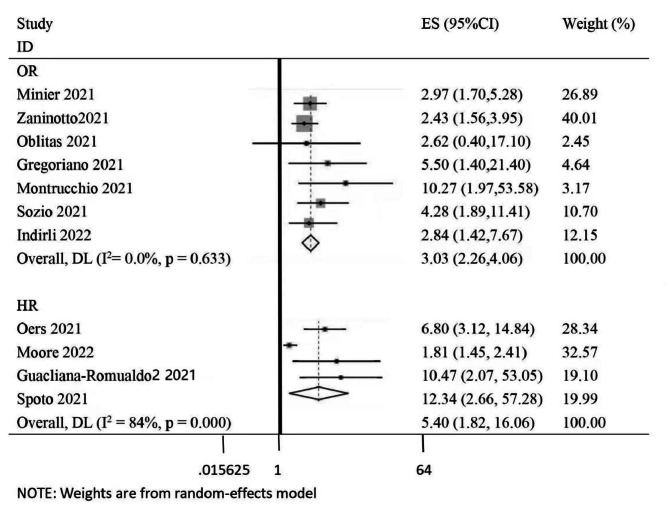
Effect size analysis for mortality in COVID-19 patients with MR-proADM elevation

We calculated an overall mortality rate of 19.0% (347/1822) for all studies, ranging from 7.2 to 54.4%. Seven studies used OR to indicate that MR-proADM was associated with mortality, and the pooled OR was 3.03 (2.26–4.06), which means that an increase of 1 nmol/L of MR-proADM was independently associated with a more than threefold increase in mortality. There was no significant heterogeneity between these studies (I^2^ = 0.0%, P = 0.633) (Fig. 6). Four studies used HR as an effect measure, and the pooled HR was 5.40 (1.82–16.06). Significant heterogeneity was observed (I^2^ = 84.0%, P = 0.000) (Fig. 6). Both of them indicated that MR-proADM can be an independent predictor for mortality among patients with COVID-19.

### Comparation with other biomarkers and critical illness scores

Some studies compared the area under the curve (AUC) of MR-proADM with other biomarkers and critical illness scores. The predictive value of MR-proADM for mortality was better than the Sequential Organ Failure Assessment (SOFA) score, Acute Physiological and Chronic Health Evaluation IV (APACHE IV), Confusion blood Urea nitrogen Respiratory rate Blood pressure age 65 or older (CURB-65) score, D-dimer, cardiac troponin T (cTNT), C-reactive protein (CRP), procalcitonin (PCT), ferritin, lactate and C-terminal proendothelin-1 (CT-proET-1) [33]. Another study found that the AUC of MR-proADM was greater than those of PCT, creatinine, albumin, platelet count, interleukin-6 (IL-6) and lymphocyte count [19]. The study of Benedetti showed that its predictive power was stronger than SOFA and Simplified Acute Physiology Score II (SAPS II), but weaker than the Acute Physiology and Chronic Health Evaluation II (APACHE II) score [17]. MR-proADM was superior to CRP, PCT, neutrophils, lymphocytes [27] and many other biomarkers [18]. However, only a few studies indicated the sensitivity and specificity, and we could not calculate the combined effect of each item.

### Sensitivity analysis and potential publication bias

**Fig. 7:**
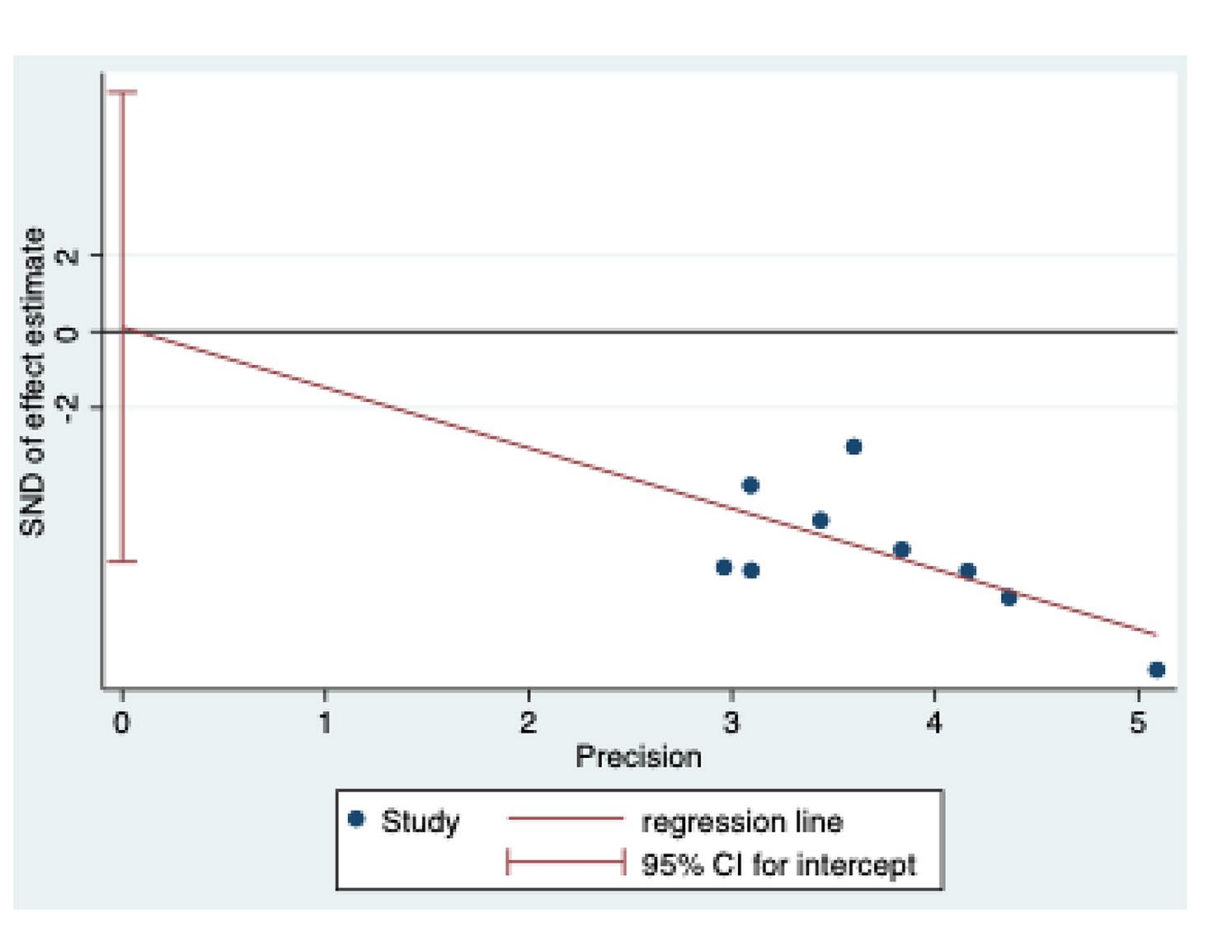
Begg’s test for publication bias

**Fig. 8:**
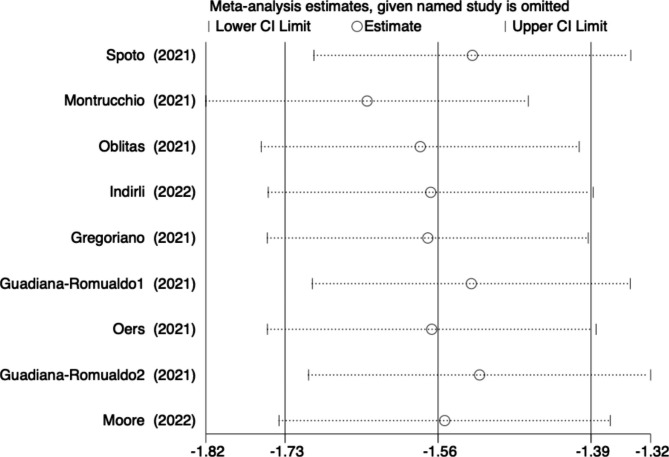
Sensitivity analysis of the mortality in COVID-19 patients with MR-proADM elevation

Begg’s test was used to assess for publication bias, and the results showed no potential bias (P = 0.965) (Fig. 7). We also conducted a sensitivity analysis, and the results indicated that our study was stable (Fig. 8).

## Discussion

Using a meta-analytic approach, we systematically reviewed and analyzed the contribution of MR-proADM to mortality in COVID-19 patients. In this study, we found that MR-proADM had a very good predictive value for the poor prognosis of COVID-19 patients. Increased levels of MR-proADM were independently associated with mortality in COVID-19 patients. These findings also confirm the feasibility of risk stratification by MR-proADM in COVID-19 patients.

COVID-19 patients have a high mortality rate. The mortality rate of in-hospital patients was 12.44% [36], that of ICU patients was 26% [37], and that of patients with acute respiratory distress syndrome (ARDS) was 50% [38]. The study population we included had different severities of illness, and the overall mortality rate was 19%. Many studies have used various biomarkers, such as CRP [39–44], PCT [39, 43, 45, 46], IL-6 [39, 43, 46], WBC [40, 47–49], D-dimer [42, 44, 46, 50, 51], lactate dehydrogenase (LDH) [39, 42–44, 46, 47], N-terminal pro-B-type natriuretic peptide (NT-proBNP) [39, 52, 53], and Troponin T [39, 54], and critical illness scores, such as APACHE II [55–57], SOFA [55, 56, 58–60], SAPS [61–64], and CURB65 [59, 61, 65], to evaluate the prognosis of patients with COVID-19. The APACHE II and SOFA scoring systems require the worst values of the clinical and biological parameters to be recorded within 24 h of admission [66]. In contrast, biomarkers can be measured rapidly within hours of the admission. WBC, CRP and IL-6 have a lack of specificity, and PCT is mainly used to determine if a bacterial infection is present and to guide the use of antibiotics [67].

Adrenomedullin (ADM) is a 52 amino acid peptide hormone that is associated with cardiovascular, endocrine and renal mechanisms regulating water and electrolyte balance [68]. ADM can reduce the permeability of capillaries during septic shock [11], and plays an important role in the regulation of inflammatory mediators, vascular endothelial barrier and microcirculation stability [11, 69]. Because of the rapid degradation and clearance of ADM in the cycle, the measurement of ADM in the cycle becomes complex [70]. Meanwhile, MR-proADM has a longer half-life and is relatively stable in the circulation, which can directly reflect the level of rapidly degraded active ADM peptide. It has been shown that measuring MR-proADM is more suitable for clinical practice [71].

In fact, patients with decreasing PCT concentrations but continuously high MR-proADM concentrations had a significantly increased mortality risk [72], and MR-proADM had an important role in predicting the development of organ failure over 24 h [73], suggesting that MR-proADM may have important clinical value in the early risk stratification of patients with sepsis. Recent studies have proposed that virus-induced endothelial dysfunction and damage, resulting in impaired vascular blood flow, coagulation and leakage, may partially explain the development of organ dysfunction [74, 75]. Another study showed that myocardial injury induced by SARS-CoV-2 is strongly associated with high MR-proADM values and mortality [76]. The assessment of MR-proADM may provide vital information to explain the pathophysiological mechanisms of vascular endothelial injury and subsequent organ dysfunction in patients with COVID-19.

Our study found that there was a significant difference in the concentration of MR-proADM between the survivors and the non-survivors (P < 0.01), the combined sensitivity was 0.86, and the combined specificity was 0.78. These findings are consistent with those of prior studies [72, 77, 78]. MR-proADM may be a good predictive marker for the prognosis of patients. Our data indicated that an increase of 1 nmol/L of MR-proADM was independently associated with a more than threefold increase in mortality (OR 3.03, 95% CI 2.26–4.06). In another meta-analysis, all of included studies showed that an elevated MR-proADM level was associated with higher risk of death from community-acquired pneumonia, the pooled RR was 5.83 (95% CI 4.53–7.52) [79]. In addition, PCT and MR-proADM test combination presents a high positive predictive value in pneumonia diagnosis and prognosis [80]. Furthermore, MR pro-ADM value provides additional information for better risk stratification especially for patients with high pneumonia severity score [81]. In a recent narrative review [82], directly testing for MR-proADM in the emergency department could contribute to improving the prognostic assessment of patients with sepsis. Spoto et al. [83] conducted a retrospective analysis of MR-proADM in 571 consecutive patients with sepsis and found that MR-proADM cut-off values > 3.39 nmol/L in sepsis and > 4.33 nmol/L in septic shock were associated with a significantly higher risk of 90-day mortality. These findings confirm that MR pro-ADM is a prognostic tool that can guide clinicians to develop more personalized treatment for patients.

Our study showed that MR-proADM was superior to most biomarkers and critical illness scores in predicting the prognosis of COVID-19 patients [17–19, 27, 33]. However, few of them indicated the sensitivity and specificity, and we could not calculate the combined effect of each item. A recent study [84] evaluated the usefulness of MR-proADM compared to CRP, PCT, and ferritin in the prognosis of influenza A (H1N1) pneumonia, and found that the initial MR-proADM, ferritin, CRP, and PCT levels effectively determined adverse outcomes and risk of ICU admission and mortality in patients with influenza virus pneumonia. MR-proADM has the highest potency for survival prediction (AUC = 0.853, P < 0.0001). The recently published TRIAGE study [85] is a multinational, prospective, observational cohort study that included consecutive medical patients presenting with a medical urgency at three tertiary-care hospitals. A total of 7,132 patients were included in the final analysis. The study found that MR-proADM was the best biomarker, especially for mortality prediction. Another study [72] indicated that the initial use of MR-proADM within the first 24 h after sepsis diagnosis resulted in the strongest association with short-term, mid-term and long-term mortality compared to all other biomarkers or clinical scores. Other studies also yielded similar results [86, 87]. Recently, a meta-analysis suggested that MR-proADM testing alone is poor at identifying invasive bacterial infections in young children [88], which may be related to the population under study, the measurement time and other factors. In addition, it may be more meaningful to determine the time trend of MR-proADM levels in septic patients with pulmonary infection [77].

There are some limitations in our study. First, selection bias and information bias are easily generated because of the observational design and different research locations. Second, there was considerable heterogeneity in some analyses, mainly due to the significant differences in the baseline patient characteristics. Third, converting the median to an average also affects our results. Due to the limited number of articles included, our analysis results may not be accurate, and more studies need to be included for verification in the future.

## Conclusions

MR-proADM was associated with mortality in patients with COVID-19, and risk assessment using MR-proADM allows clinicians to identify patients with COVID-19 who are at higher risk of adverse clinical outcomes.

## Electronic supplementary material

Below is the link to the electronic supplementary material.

Supplementary Material 1

## Data Availability

The data that support the findings of this study are included in this published article and its supplementary material.

## Abbreviations

COVID-19: coronavirus disease 2019
SARS-CoV-2: Severe Acute Respiratory Syndrome Coronavirus 2
MR-proADM: Mid-regional Proadrenomedullin
PRISMA: the Preferred Reporting Items for Systematic Reviews and Meta-Analyses
CNKI: Chinese National Knowledge Infrastructure
QUADAS-2: The Quality Assessment of Diagnostic Accuracy Studies
SD: standard deviation
WMD: weighted mean difference
CI: confidence interval
IQR: interquartile range
ICU: intensive care unit
IMCU: intermediate medical care unit
NHS: national health service
AUC: the area under the curve
SOFA: the Sequential Organ Failure Assessment
APACHEIV: Acute Physiological and Chronic Health Evaluation IV
CURB-65: Confusion blood Urea nitrogen Respiratory rate Blood pressure age 65 or older
cTNT: cardiac troponin T
CRP: C-reactive protein
PCT: procalcitonin
CT-proET-1: C-terminal proendothelin-1
IL-6: interleukin-6
SAPS II: Simplified Acute Physiology Score II
APACHE II: the Acute Physiology and Chronic Health Evaluation II score
ARDS: acute respiratory distress syndrome
LDH: lactate dehydrogenase
NT-proBNP: N-terminal pro-B-type natriuretic peptide
ADM: Adrenomedullin
