# Supplementary material for: Circulating mid-regional proadrenomedullin is a predictor of mortality in patients with COVID-19: a systematic review and meta-analysis
Source: BMC Infect Dis. 2023 May 8;23:305. doi: 10.1186/s12879-023-08275-z (PMC10165584; doi:10.1186/s12879-023-08275-z)
Supplement: Supplementary file 1 — Supplementary Material 1 [file 12879_2023_8275_MOESM1_ESM.docx]

## Additional file

Table 1 A summary of the reported literature for MR-proADM in COVID-19 patients

| **Author, year** | **Country** | **Study periods** | **Study design** | **Setting** | **Population** | **Sample size** | **Male, n(%)** | **Age(Mean±SD/Median,range,years)** | **Clinical outcomes** |
| --- | --- | --- | --- | --- | --- | --- | --- | --- | --- |
| Sasso, 2021 | Italy | from September to October 2020 | RS, SC | NA | patients with COVID-19 | 110 | 61 (55) | 62 (52-76) | Hospital discharge, ICU transfer, In-hospital mortality |
| Minier, 2021 | Italy | NA | PS, SC | Emergency Department | patients with COVID-19, age>18 | 321 | 215 (67) | 63.3±14.7 | In-hospital mortality |
| Zaninotto, 2021 | Italy | Between November 12th and 24th 2020 | RS, SC | Hospital | patients with COVID-19 | 135 | 100 (74) | 67 (58-77) | Discharge, In-hospital mortality |
| Oers, 2021 | Netherlands | from March 11 until May 27, 2020 | PS, SC | ICU | critical ill patients with COVID-19 | 105 | 80 (76) | 68 (59-74) | 28-day mortality |
| Guadiana-Romualdo1, 2021* | Spain | between September and October 2020 | PS, MC | Emergency Department | patients with COVID-19, age≥14 | 359 | 230 (64.1) | 59 (47-71) | 90-day mortality |
| Oblitas, 2021 | Spain | between August and November 2020 | PS, SC | ICU | critical ill patients with COVID-19， age≥18 | 95 | 64 (67.4) | 60.3±12.9 | 30-day mortality |
| Spoto, 2021 | Italy | between 1st April and 30th June 2020 | PS, SC | COVID Center | patients with COVID-19 | 69 | 37 (53.6) | 78 (61-84) | ARDS development, 30-day mortality |
| Gregoriano, 2021 | Switzerland | between February and April 2020. | PS, SC | Hospital | patients with COVID-19，age≥18 | 89 | 58 (65) | 67 (58-74) | In-hospital mortality |
| Montrucchio, 2021 | Italy | between March and June 2020 | PS, SC | ICU | patients with COVID-19，age≥18 | 57 | 50 (87.7) | 64 (54-71) | 28-day mortality |
| Moore, 2022 | UK | between April and June 2020 | RS, SC | Acute HNS Setting | patients with COVID-19 | 135 | 70 (51.9) | 64.6±19.5 | 30-day mortality |
| Sozio, 2021 | Italy | from 25th March to 15th May 2020 | RS, SC | Hospital | patients with COVID-19 pneumonia | 111 | 66 (59.5) | 62.3±13.6 | Death and orotracheal,  intubation during hospitalization |
| Guadiana-Romualdo2, 2021* | Spain | between March and April 2020 | PS, MC | Emergency Department | patients with COVID-19 | 99 | 61 (61.6) | 66±15 | 28-day mortality |
| Benedetti, 2021 | Italy | between March 2020 and April 2020 | PS, SC | IMCU | patients diagnosed with ARDS related to COVID-19 | 21 | 15 (71.4) | 70.9 (54-85) | 30-day mortality |
| Indirli, 2022 | Italy | between March and June 2020 | RS, SC | Medium intensity-of-care COVID-19 Department | patients with COVID-19，age≥18 | 116 | 65 (56) | 66±15 | In-hospital mortality |
|  | | | | | | | | | |
| * same name but different study. RS, retrospective studies; PS, prospective study; SC, sing-center; MC, multicenter; NHS, national health service; IMCU, intermediate medical care unit; ICU, intensive care unit. | | | | | | | | | |

Table 2 A summary of clinical studies of MR-proADM in COVID-19 patients according to survival outcomes

| Author, year | MR-proADM(nmol/L) | | | Mortality rate, n(%) |
| --- | --- | --- | --- | --- |
|  | All | Non-survivors | Survivors |  |
| Sasso, 2021 | 0.93 (0.58-1.09) | 2.59 (2.3-2.95) | 0.82 (0.57-1.03) | 14 (13) |
| Minier, 2021 | NA | NA | NA | 97 (30) |
| Zaninotto, 2021 | 0.93 (0.64-1.46) | NA | NA | 14 (10.4) |
| Oers, 2021 | 1.16 (0.85-1.71) | 1.88 (1.35-2.64) | 1.01 (0.80-1.28) | 30 (28.6) |
| Guadiana-Romualdo1, 2021* | 0.76 (0.57-1.15) | 1.50 (0.98-2.31) | 0.73 (0.56-1.06) | 32 (8.9) |
| Oblitas, 2021 | 0.77 (0.61-1.14) | 1.22 (0.84-2.33) | 0.76 (0.6-1.03) | 12 (12.6) |
| Spoto, 2021 | 1.49 (0.67-2.26) | 5.25 (2.67-6.53) | 1.15 (0.57-1.85) | 16 (23.2) |
| Gregoriano, 2021 | NA | 1.3 (1.1-2.3) | 0.8 (0.7-1.1) | 17 (19) |
| Montrucchio, 2021 | NA | 2.65±2.33 | 1.18±0.47 | 31 (54.4) |
| Moore, 2022 | 1.02 (0.71-1.61) | 1.842 (1.3943-2.6323) | 0.899 (0.6284-1.316) | 30 (22.2) |
| Sozio, 2021 | 0.82 (0.64-1.08) | 1.38 (0.94-1.73) | 0.73 (0.56-0.94) | NA |
| Guadiana-Romualdo2, 2021* | 0.74 (0.60-1.02) | 1.54 (1.05-2.12) | 0.68 (0.57-0.94) | 14 (14.1) |
| Benedetti, 2021 | 2.3±2.7 | 3.5 | 1.1 | 11 (52.4) |
| Indirli, 2022 | 0.9 (0.6-1.3) | 1.5 (1.1-2.8) | 0.8 (0.6-1.1) | 21 (18) |

* same name but different study.

Table 3 Main studies and findings on the prognostic role of MR-proADM concentration in COVID-19 patients

| Author, year | AUC for death | Cut-off (nmol/L) | Sens% | Spec% | Results |
| --- | --- | --- | --- | --- | --- |
| Sasso, 2021 | 0.95 | 1.73 | 90 | 95 | MR-proADM was significantly associated with in-hospital mortality (r=0.64, 95%CI 0.45-0.78, P<0.001) |
| Minier, 2021 | 0.85 | 1.105 | NA | NA | Patients with a value of MR-proADM higher than the cut-off value of 1.105 show a threefold increase in mortality (OR 2.97, CI 1.7-5.28) |
| Zaninotto, 2021 | 0.9 | NA | NA | NA | MR-proADM was the most significant predictor for in-hospital death (OR 2.43, CI 1.56-3.95) |
| Oers, 2021 | 0.84 | 1.57 | 88 | 67 | MR-proADM ≥1.57nmol/L was significant predictor for 28-day mortality with high hazard ratio (HR 6.80, 95% CI 3.12-14.84) |
| Guadiana-Romualdo1, 2021* | 0.832 | 0.8 | 96.9 | 58.4 | Circulating MR-proADM levels (inverse transformed), after adjusting by a propensity score including eleven potential confounders, were an independent predictor of 90-day mortality (HR 0.162, 95%CI 0.043-0.480) |
| Oblitas, 2021 | 0.73 | 1.0 | 66.7 | 72.3 | Univariate logistic regression analysis showed that MR-proADM level ≥1nmol/L was associated with mortality (crude OR 5.22, CI 1.42-19.1, P=0.013). However, multivariate logistic regression analysis showed that MR-proADM levels ≥1nmol/L was not independently associated with mortality (adjusted OR 2.62, 95%CI 0.4-17.1, P=0.314) |
| Spoto, 2021 | 0.698 | 2.0 | 78.6 | 88 | Patients presenting with MR-proADM values ≥ 2 nmol/L showed a significantly higher mortality risk than patients with MR-proADM values < 2 nmol/L (adjusted HR 12.34; 95% CI 2.66-57.28） |
| Gregoriano, 2021 | 0.78 | 0.93 | 92.9 | 60 | An increase of 1 nmol/L of admission MR-proADM was independently associated with a more than fivefold increase in in-hospital mortality (adjusted OR 5.5, 95% CI 1.4–21.4, P=0.015). |
| Montrucchio, 2021 | 0.85 | 1.8 | NA | NA | MR-proADM>1.8nmol/L was a significant predictor for ICU mortality (OR 10.272, 95% CI 1.970-53.578) |
| Moore, 2022 | 0.8441 | 1.54 | NA | NA | MR-proADM had the greatest ability to predict 30-day mortality (HR 1.809, 95% CI 1.454-2.407) |
| Sozio, 2021 | 0.849 | 0.895 | 85.7 | 68.7 | High MR-proADM levels were significantly associated with negative outcomes (death or orotracheal intubation),with an OR of 4.284 (1.893-11.413) |
| Guadiana-Romualdo2, 2021* | 0.905 | 1.01 | 85.7 | 84.7 | MR-proADM plasma levels above optimal cut-off (1.01nmol/L) showed the strongest independent association with 28-day mortality risk (HR 10.470, 95% CI 2.066-53.049, P<0.005) |
| Benedetti, 2021 | 0.81 | 1.07 | 91 | 71 | MR-proADM could be useful to predict outcomes in COVID-19 ARDS patients |
| Indirli, 2022 | 0.79 | 1.0 | 71.3 | 85.7 | In multivariable analysis, mortality resulted significantly associated with MR-proADM (adjusted OR 2.844, 95%CI 1.421-7.671, P=0.01) |

* same name but different study. OR, odds ratio; HR, hazard ratio; CI, confidence interval.
